# Salivary Glands after Prolonged Aluminum Exposure: Proteomic Approach Underlying Biochemical and Morphological Impairments in Rats

**DOI:** 10.3390/ijms23042251

**Published:** 2022-02-18

**Authors:** Deiweson Souza-Monteiro, Márcia Cristina dos Santos Guerra, Leonardo Oliveira Bittencourt, Walessa Alana Bragança Aragão, Aline Dionizio, Felipe Martins Silveira, Marília Afonso Rebelo Buzalaf, Manoela Domingues Martins, Maria Elena Crespo-Lopez, Rafael Rodrigues Lima

**Affiliations:** 1 Laboratory of Functional and Structural Biology, Biological Sciences Institute, Federal University of Pará, Belém 66075-110, Brazil; deiweson.monteiro@gmail.com (D.S.-M.); marciacsguerra@gmail.com (M.C.d.S.G.); leo.bittencourt25@gmail.com (L.O.B.); walessa.aragao@gmail.com (W.A.B.A.); 2 Department of Biological Sciences, Bauru School of Dentistry, University of São Paulo, Bauru 17012-901, Brazil; alinesdionizio@usp.br (A.D.); mbuzalaf@fob.usp.br (M.A.R.B.); 3 Faculty of Department of Oral Pathology, School of Dentistry, Federal University of Rio Grande do Sul, Porto Alegre 90040-060, Brazil; fp.martinss@gmail.com (F.M.S.); manomartins@gmail.com (M.D.M.); 4 Laboratory of Molecular Pharmacology, Institute of Biological Sciences, Federal University of Pará, Belém 66075-110, Brazil; maria.elena.crespo.lopez@gmail.com

**Keywords:** aluminum, salivary glands, oxidative stress, proteomic, morphology, toxicology, intoxication

## Abstract

Aluminum (Al) is one of the most abundant elements on Earth, and its high extraction rate and industrial use make human exposure very common. As Al may be a human toxicant, it is important to investigate the effects of Al exposure, mainly at low doses and for prolonged periods, by simulating human exposure. This work aimed to study the effects of low-dose exposure to chloride aluminum (AlCl_3_) on the oxidative biochemistry, proteomic profile, and morphology of the major salivary glands. Wistar male rats were exposed to 8.3 mg/kg/day of AlCl_3_ via intragastric gavage for 60 days. Then, the parotid and submandibular glands were subjected to biochemical assays, proteomic evaluation, and histological analysis. Al caused oxidative imbalance in both salivary glands. Dysregulation of protein expression, mainly of those related to cytoarchitecture, energy metabolism and glandular function, was detected in both salivary glands. Al also promoted histological alterations, such as acinar atrophy and an increase in parenchymal tissue. Prolonged exposure to Al, even at low doses, was able to modulate molecular alterations associated with morphological impairments in the salivary glands of rats. From this perspective, prolonged Al exposure may be a risk to exposed populations and their oral health.

## 1. Introduction

Aluminum (Al) is an element that is widely distributed in the Earth’s crust and is of great environmental importance because of its excellent physical properties; thus, it is used by industries at large scales [1]. The metallic form of Al is not directly found in soil, so its extraction is dependent on the mining of Bauxite, which is the primary mineral source of Al [2,3].

Humans are exposed to Al by drug consumption, cosmetics, food, and Al-enriched water [4,5,6]. The presence of residual Al in drinking water is one of the most common forms of exposure to the metal [7] and studies indicate that Al can enter the human body through a combination of Al ions with other molecules, forming aluminum salts that can be easily absorbed in the stomach [8,9]. Then, Al is distributed throughout the organism and can affect many organs, such as the cerebellum [10], kidneys [11] and even the salivary glands [12] of animals.

Salivary glands are important components of the stomatognathic system since they synthesize and excrete saliva. Saliva performs several functions: protection and lubrication of the oral tissues, initiation of digestion, exertion of antibacterial effects, tissue repair, and buffering [13]. Malfunction of the salivary glands can interfere with oral homeostasis by altering the quality and quantity of saliva, which can cause or aggravate several disorders, such as xerostomia and periodontal disease [14,15]. Studies from our group have already shown that salivary glands are susceptible to injury caused by exposure to metals, such as mercury, lead, and even Al [16,17,18,19,20,21,22].

In previous studies from our group, we showed Al deposition in the salivary glands [12] and also oxidative stress and morphology impairment after low-dose exposure to Al [22]. However, little is known about the mechanisms related to the effects of chronic and low-dose Al exposure, especially the changes related to molecular impairments.

From this perspective, this study aimed to investigate the effects of low-dose and long-term exposure to Al, in the salivary glands of rats, as a means of simulating human exposure. Our study evaluated the possibility of oxidative imbalance and its association with the modulation of the proteomic profile and impairments in the morphology of the two main pairs of major salivary glands after chronic Al exposure.

## 2. Results

### 2.1. Al Triggered Biochemical Changes Related to Redox Homeostasis in the Salivary Glands of Rats 

**Figure 1:**
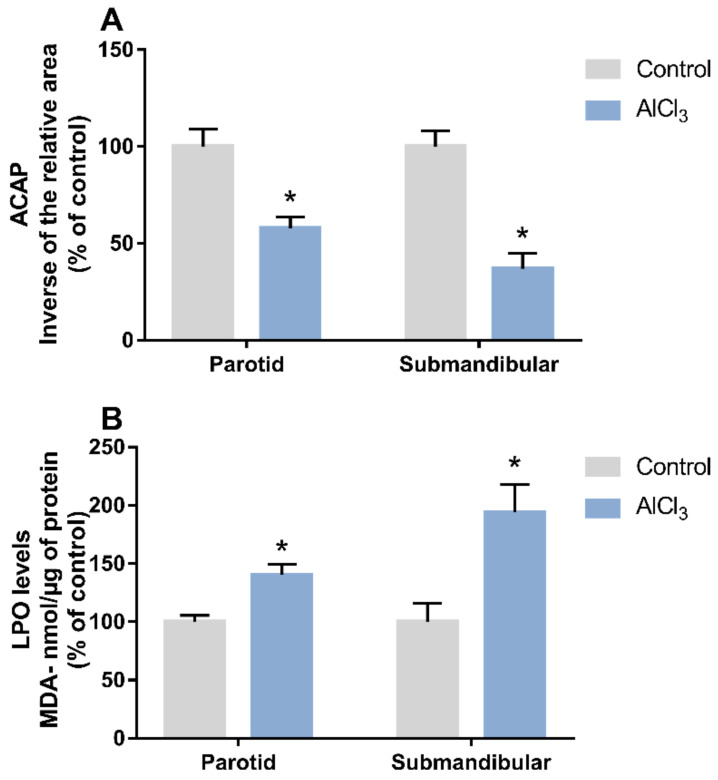
Effects of exposure to Aluminum Chloride (AlCl_3_) (8.3 mg/kg/day) for 60 days on the oxidative biochemistry of salivary glands. (**A**) Antioxidant capacity against peroxyl radicals (ACAP). (**B**) Lipid peroxidation (LPO) levels. The results are expressed as the mean ± standard error of the mean (SEM). Student’s *t*-test, * *p* < 0.05.

For this parameter, the antioxidant capacity against peroxyl radicals (ACAP) was tested, in which the ROS levels in the samples were measured. These were subjected to two readings in a fluorimeter: samples that received the addition of a peroxyl radical generator and samples without this addition (blank samples). Total fluorescence over time was fitted to a polynomial function that generated area graphs with and without radical generation. The relative area was quantified and for better expression of the data in bar graphs, the inverse of the relative area was used. Therefore, our results show that exposure to Al reduced the ACAP in the parotid glands (aluminum chloride (AlCl_3_): 57.79 ± 5.73%; control: 100 ± 8.99%, *p* = 0.0019) and in the submandibular glands (AlCl_3_: 36.9 ± 7.94%; control: 100 ± 8.17%, *p* = 0.0001) (Figure 1A). 

In addition, the lipid peroxidation (LPO) assay was performed, where the levels of the malondialdehyde (MDA) metabolite were measured. Thus, long-term exposure to Al increased the LPO levels in the parotid glands (AlCl_3_: 140.4 ± 9.01%; control: 100 ± 5.61%, *p* = 0.0018) and in the submandibular glands (AlCl_3_: 194.1 ± 23.9%; control: 100 ± 15.89%, *p* = 0.0187) (Figure 1B).

### 2.2. Long-Term AlCl_3_ Exposure Promoted Significant Changes in the Global Proteomic Profile of Major Salivary Glands 

A total of 372 proteins showed different regulation statuses in the parotid glands of rats. Among these proteins, 22 proteins were uniquely identified in the control group and 220 in the exposed group. In addition, the expression of 39 proteins was downregulated, and the expression of 91 was upregulated.

Regarding the submandibular proteome, a total of 63 proteins with different regulation statuses were identified. Among these, 49 proteins were uniquely identified in the control group and one protein was uniquely identified in the aluminum group. In a quantitative analysis between the groups, the expression of nine proteins was downregulated and the expression of four proteins was upregulated. The complete data are available in Tables S1 and S2.

**Figure 2:**
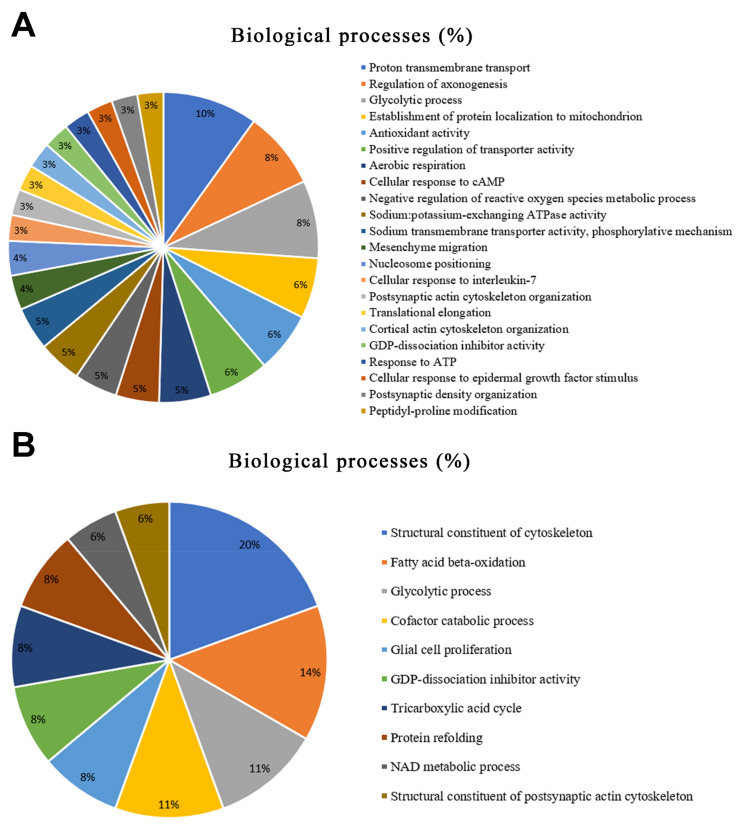
Functional distribution of proteins identified with different expression (**A**) in the parotid glands and (**B**) in the submandibular glands of rats exposed to AlCl_3_ vs. control. Categories of proteins are based on Gene Ontology annotation of the biological process. Protein accession numbers were provided by Uniprot.org, accessed on 11 November 2021. The gene ontology was evaluated according to the ClueGo plugin of Cytoscapes software 3.6.

According to Gene Ontology, the modulated proteins are related to 19 biological processes in the parotid glands. The top five most affected processes were proton transmembrane transport (10%), followed by the regulation of axonogenesis (8%), the glycolytic process (8%), the establishment of protein localization to mitochondria (6%), and antioxidant activity (6%) (Figure 2A).

In the submandibular glands, 10 categories of biological processes were affected by aluminum exposure, of which the top five were structural constituents of the cytoskeleton (20%), followed by fatty acid beta-oxidation (14%), the glycolytic process (11%), the cofactors of the catabolic process (11%), and glial cell proliferation (8%) (Figure 2B).

**Figure 3:**
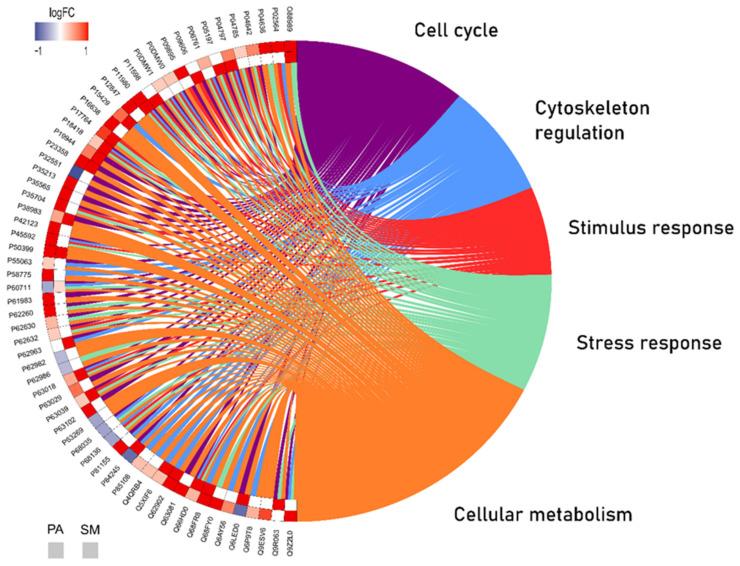
Over-representation analysis (Circos plot) presenting the protein–protein interactions between the aluminum and control groups in both parotid (PA; the outside square) and submandibular (SM; the inner square) glands and the biological processes involved according to Gene Ontology. The altered regulation is expressed according to the colors. Blue represents subexpression, red represents superexpression and white represents absence.

Based on the proteomic profile analysis, the data were compiled to evaluate the protein–protein interaction (PPI), the unique and different expression of the experimental groups in each salivary gland and the related biological functions with the Circos plot tool, an over-representation analysis (ORA). Thus, it was possible to observe the interactions of 60 proteins present in both glands considering their up/downregulation or absence between the control vs. aluminum group and the main biological processes involved (Figure 3). 

### 2.3. Salivary Gland Morphology Was Affected by Chronic AlCl_3_ Exposure

Histopathological analysis of the parotid glands revealed multiple serous acinus arranged in lobules, while in submandibular glands, the serous acinus was associated with mucous tubulous. Some major morphological differences were observed when comparing parotid and submandibular glands in both the control and AlCl_3_ groups. In the parotid glands, it was observed that the AlCl_3_ group exhibited smaller size of the acinus and a greater amount of stroma than the control group. In the submandibular glands, the AlCl_3_ group exhibited more stromal components and larger ductal structures than the control group. In addition, the size of the acinus from the AlCl_3_ group also appeared smaller than that of the control group. No inflammatory components were present in the analyzed glands.

**Figure 4:**
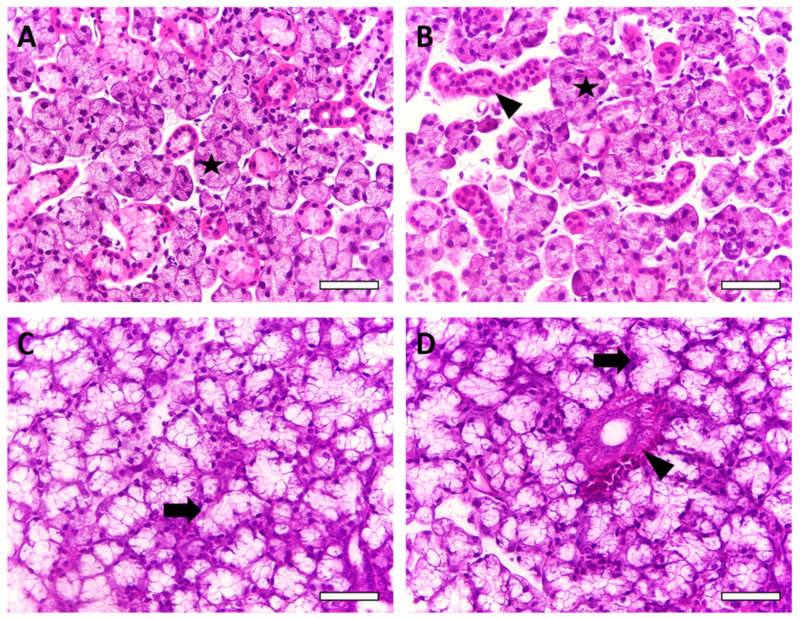
Effects of long-term AlCl_3_ exposure (8.3 mg/kg/day) on rat salivary glands. Representative photomicrographs of (**A**) control and (**B**) aluminum parotid glands and (**C**) control and (**D**) aluminum submandibular glands. Stars indicate serous acinus; arrows indicate mucous tubulous and arrowheads indicate ducts. The sections were stained with hematoxylin and eosin (H&E). Scale bar = 50 µm.

After the morphometric measurements, it was found that in the parotid glands, the tissue of the parenchyma decreased (*p* = 0.0397), while the stromal tissue increased (*p* = 0.0011). The total acinar area also decreased after 60 days of exposure to Al (*p* = 0.0326), but there were no significant changes in the total area of the ducts (*p* = 0.7852). A decrease in the submandibular gland’s parenchyma area (*p* = 0.0001) was also observed, while the stromal tissue increased in the exposed group (*p* < 0.0001). In addition, the total acinar area decreased in the AlCl_3_ group compared to the control group (*p* = 0.0006). An increase in the total area of the ducts was observed in the AlCl_3_ group (*p* = 0.0312) (Table 1, Figure 4).

## 3. Discussion

Our study provides new evidence about the effects of AlCl_3_ on the major salivary glands of rats after long-term exposure at a dose equivalent to those which humans are exposed via dietary levels. In a previous study, Al was found to have harmful effects on the oxidative state and the morphology of salivary glands [22]. For a better understanding about the molecular state and how Al interacts in those organs, our study presents an unprecedented global proteomic investigation showing that long-term exposure to Al affects several biological processes in the salivary glands, such as energy metabolism, the cytoskeleton, and cell proliferation, and it is associated with oxidative biochemistry imbalance, which was found to culminate in a severe morphological impairment in both glands. 

Our study used a low dose that was described in a previous study as being able to promote changes in the central nervous system [10] and inperipheral organs [23] of rats. This dose was established considering the provisional acceptable weekly intake of Al by humans, which ranges from 1 to 2 mg/kg body weight in accordance to the joint FAO/World Health Organization Expert Committee on Food Additives [24], and an allometric calculation that considers the biological differences between rodents and humans, such as the body surface area of the species, which influences blood volume, caloric expenditure, and renal functions [23,25]. 

To ensure Al consumption, AlCl_3_ was administered by orogastric gavage. In previous studies, this method triggered harmful effects in the central nervous system [10] and in other oral structures such as alveolar bone [26], and the success of this method was validated by the quantification of the blood concentration of Al. In those cited studies, the animals exposed to AlCl_3_ exhibited higher levels of Al in the blood in comparison with the non-exposed animals, without significant alterations in weight and mortality between the groups. 

Our group has performed several studies regarding the effects of metals on the salivary glands in animal models, showing that those elements are capable of trigger tissue and redox impairments that affect the animals’ stomatognathic system [16,17,18,19,20,21,22]. Other studies have also revealed damage to those organs after Al exposure [27,28], but to the best of our knowledge, this is the first study that used a proteomic approach to investigate the effects of exposure to a translational dose of Al over salivary glands.

The mechanisms underlying Al are still not fully understood. One study gave evidence suggesting that Al is involved in oxidative stress conditions and may exert significant pro-oxidant activity [29]. This was suggested because Al^3+^ was previously reported to bind to the superoxide anion radical (O_2_¯•, superoxide) to form the Al superoxide semireduced radical ion (AlO_2_^2+^•, Al-superoxide complex) [30]. Computational approaches showed that these Al-superoxide complexes reduce Fe(III) to Fe(II) in Fenton’s reaction, releasing oxygen molecules and generating ROS [31]. Increased reactive oxygen species (ROS) production and decreased antioxidant activity are the main factors leading to the imbalance between the oxidant and antioxidant systems and, consequently, oxidative stress [32]. In our study, the antioxidant competence, ACAP, was significantly reduced in both parotid and submandibular glands after exposure to Al (Figure 1A). A decrease in ACAP was previously observed in the central nervous system of rats exposed to Al [33] and our data show that other organs, such as salivary glands, can also be susceptible to changes in the antioxidant system in accordance with the existing literature on Al toxicology. 

LPO levels were increased in both glands after Al exposure (Figure 1B). ROS are highly reactive and attack various classes of molecules including proteins, DNA, and lipids such as polyunsaturated fatty acids [34]. When the polyunsaturated fatty acid chain is oxidized, MDA and other molecules, such as 4-hydroxy-2-nonenal and F2-isoprostanes, are formed from this reaction [35]. These molecules are widely accepted biomarkers of oxidative stress because of its genotoxicity or cytotoxicity. In a previous study by our group, the levels of LPO, as well as ACAP levels, on salivary glands of mice were affected by Al exposure and those changes were also related to morphometry impairments [22]. In another study, AlCl_3_ caused increased LPO levels in the liver and kidneys of adult rats and also reduced levels of other antioxidant molecules such as superoxide dismutase and catalase, thereby causing oxidative stress [36]. Our data emphasize that oxidative stress may be one of the toxic mechanisms of Al.

Previous reports have shown that major salivary glands are susceptible to metal-induced toxicity in models of exposure to lead [21], inorganic mercury [16], and methylmercury [17,18,19]. The last model also induces several proteomic damages, in the parotid and submandibular glands, especially changes in proteins related to cytoskeleton and energy metabolism pathways [17]. The bioinformatic analysis of the present study showed biological processes related to mitochondrial activity in the parotid gland and protein refolding in the submandibular gland, and these findings are suggestive of not only energy metabolism impairment but also oxidative stress pathway activation and proteome protection impairment.

The heat shock proteins (HSP) family performs important homeostasis functions, and alterations in the expression of these proteins may be related to protein impairments in the organ of interest. HSPs have chaperone activity and have already been suggested as therapeutic strategies for many diseases due to their protective functions [37]. In our study, we observed upregulation of the expression of ‘Heat shock 70 kDa protein 1A’ (P0DMW0), ‘Heat shock 70 kDa protein 1B’ (P0DMW1), ‘Heat shock 70 kDa protein 1-like’ (P55063), and ‘Heat shock cognate 71 kDa protein’ (P63018) in the parotid glands, in addition to upregulation of ‘60 kDa heat shock protein, mitochondrial’ (P63039) expression in both glands. HSP expression alteration was also observed in hippocampal neurodegeneration after metal exposure, specifically methylmercury [38]. This HSP upregulation after Al exposure may reflect a cellular response that attempts to restore the system following oxidative stress in the salivary glands.

In addition, mitochondria are responsible for the redox state and mitochondrial failure is directly involved in changes in energy metabolism and oxidative stress [39]. Studies have shown that Al has the potential to disrupt the antioxidant defense system in different regions in rat brain [10,27,33]. AlCl_3_-induced oxidative stress was shown to modulate cytochrome c in the rat hippocampus, and the release of subunits of this protein is associated with apoptosis caused by activation of the caspase cascade [27,40]. In our study, mitochondrial changes were observed in the parotid glands, as indicated by the upregulation of the expression of ‘Cytochrome c oxidase subunit 4 isoform 1, mitochondrial’ (P10888) and ‘Cytochrome c oxidase subunit 5A, mitochondrial’ (P11240). Cytochrome c was also shown to be altered in liver dysfunction and mitochondrial energy metabolism disorder in rats exposed to AlCl_3_ [41].

Moreover, calcium homeostasis is also important to mitochondria. Excessive influx of calcium (Ca^2+^) into this organelle generates more ROS and impairs mitochondrial membrane potential [42]. In the salivary glands exposed to Al, the expression of several proteins related to calcium homeostasis was upregulated in the submandibular glands; these proteins included ‘Superoxide dismutase [Cu-Zn]’ (P07632), ‘Voltage-dependent anion-selective channel protein 1′ (Q9Z2L0), and ‘Peroxiredoxin-2 (P35704)’ in the parotid glands, and ‘Peroxiredoxin-5, mitochondrial’ (Q9R063) in the submandibular glands. The upregulation these molecules could be related to more calcium influx increasing ROS and oxidative stress.

Our ORA analysis revealed the connections between the proteins in a PPI network, showing the most relevant biological processes involved. This study provides a better understanding of possible proteins related to mechanisms affecting both glands. The expression of the tubulin protein family, represented by the ‘Tubulin alpha-4A chain’ (Q5XIF6) and the ‘Tubulin alpha-3 chain’ (Q68FR8), was upregulated in both the parotid and submandibular glands from the Al-exposed animals. This group of proteins is the major component of microtubules, which support cells in many processes, including cell movement and mitosis [43]. ‘Tubulin beta-3 chain’ (Q4QRB4) was reported as being a possible biomarker of many ageing cells such as melanocytes and keratinocytes [44] and the expression of this protein and that of other isoforms of the tubulin family was upregulated in the parotid glands exposed to Al. On the other hand, tubulin isoforms were expressed exclusively in the exposed group of submandibular glands, which may be associated with the most altered biological process, the structural constituent of the cytoskeleton (20%), according to the Gene Ontology (Figure 2B).

Similar to the tubulin family, other proteins are involved in the cellular metabolism and cell cycle of the salivary glands and are also upregulated in both glands, as shown in Figure 3. Malate dehydrogenase plays a role in cellular metabolism as the enzyme that catalyzes the conversion of oxaloacetate and malate utilizing the NAD/NADH coenzyme [45]. In eukaryotic cells, at least two forms of this enzyme can be observed. One isoform is a major enzyme of the citric acid cycle that operates within mitochondria, and the other is found in the cytosol [46]. The expression of the cytoplasmic isoform (O88989) was upregulated in both salivary glands after Al exposure. In the parotid gland, greater changes were identified in the biological process of proton transmembrane transport (10%) (Figure 1A), which is related to changes in the activity of Na,K-ATPase enzymes. In turn, these proteins (P54708, Q64541) were upregulated in the group exposed to Al. These findings confirm the hypothesis that Al can affect salivary glands by changing the expression of important proteins related to the biochemical mechanisms responsible for the maintenance of cells. All these proteins could possibly be biomarkers of Al toxicity in salivary glands.

Actin is the major constituent of the microfilament apparatus in all eukaryotic cells and plays a crucial role in the cytoskeleton, especially in cell motility and muscle contraction [47]. In our study, the group of proteins related to the structural constituent of the cytoskeleton was the biological process group that was most affected in the submandibular glands (Figure 2B). Myoepithelial cells are contractile cells that are associated with the secretory cells of salivary glands, and are rich in actin proteins [48]. Myoepithelial cell contraction helps to expel saliva to the duct system in the mouth where it participates in lubrication and the initiation of digestion [49]. In both salivary glands exposed to Al, the expression of ‘Actin, gamma-enteric smooth muscle’ (P63269) and ‘Actin, cytoplasmic 1’ (P60711) was dysregulated. Histological studies have already shown a decrease in smooth muscle actin immunostaining in the salivary glands of animals after lead exposure [21] and chronic stress [50]. Alterations in the expression of these proteins could be indicative of an impairment of the nervous stimulus transmission of myoepithelial cells affecting the salivary glands after exposure to Al, which may be one of the routes of the toxic action for this metal.

Changes in actin expression were also associated with collagen accumulation in liver fibrosis due to the activation of the Rho pathway [51]. In our study, the expression of the ‘Rho GDP dissociation inhibitor’ (P50399) was upregulated in both salivary glands, which may be related to the histological findings in the salivary glands after exposure to Al. In the histopathological analysis, a greater amount of stroma in the parotid glands and more stroma components in the submandibular glands were observed on the Al group. This suggests that metal exposure could promote a change in mesenchymal cells’ metabolism. Another protein related to cell proliferation is ‘60S acidic ribosomal protein P1′ (P19944) and its expression was upregulated in the parotid and submandibular glands after exposure to Al. Elevated expression of this ribosomal protein is associated with cell proliferation and invasion in several pathologies [52]. These proteome alterations support the hypothesis that cells react by undergoing molecular changes and try to respond to the harmful effects on tissues caused by Al intoxication.

In addition, the histological quantitative parameters revealed that in both glands, exposure to Al could change the ratio of the parenchyma area to the stroma area in the gland tissue (Table 1, Figure 4), indicating the loss of the epithelial component with reparative conjunctive tissue formation. Impairments in epithelial–mesenchymal interactions are associated with the development of pathological processes, such as fibrosis, in salivary glands [53]. Moreover, in the morphometric analysis, the salivary gland structures, mainly the submandibular glands, were also affected by Al exposure. The acinar area was reduced in both salivary glands, while the ductal area was increased in the submandibular glands (Table 1). Those morphometry changes were already observed in the salivary glands of rats exposed to methylmercury [20]. Acini is the gland structure where saliva is produced, and alterations in its morphology may impair the production of this important fluid [54]. Increased ductal area may be a cellular response for overcoming possible damage through regeneration mechanisms developed by granular and intercalated ducts [55]. The different responses in duct morphology seen in the parotid and submandibular glands may be explained by the different physiological and histological effects observed in the glands as they are two independent organs [55].

The parotid glands are predominantly formed by serous acinar cells that secrete aqueous saliva, which is rich in water and ions. The submandibular glands are composed of mixed acini with mucous and serous components that produce saliva that is rich in mucin and glycoconjugates [56]. Their duct systems are also different. The intercalated ducts are longer in the parotid glands than in the submandibular glands. The ducts are the structures responsible for transporting and modifying water and electrolyte concentrations, so differences in the duct system structures between the glands also affect the final saliva production [55]. Those characteristics may be the reason why each gland can develop different responses to Al intoxication.

## 4. Materials and Methods

### 4.1. Ethics Statement and Experimental Groups

**Figure 5:**
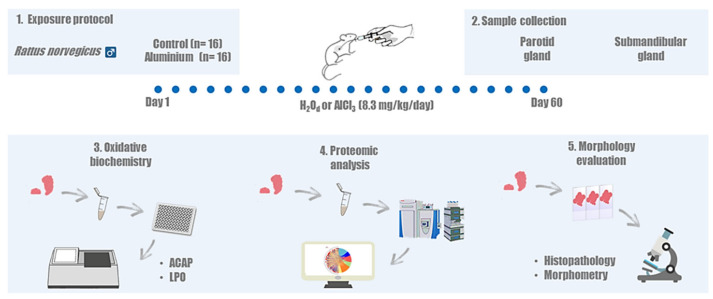
Methodological steps of the present study. (**1**) Description of experimental groups and exposure to aluminum chloride (AlCl_3_) or distilled water (H_2_Od). (**2**) Collection of the biological samples. (**3**) Oxidative biochemistry assays performed by measuring the antioxidant capacity against peroxyl radicals (ACAP) and lipid peroxidation (LPO) levels. (**4**) Proteomic profile analysis performed by mass spectrometry system and (**5**) morphology analysis.

This study was conducted after approval by the Ethics Committee on Experimental Animals of Federal University of Pará (under license number 5923210617), following the Guide for the Care and Use of Laboratory Animals [57]. This study followed the Animal Research: Reporting of In Vivo Experiments (ARRIVE) guidelines [58]. The sample size was calculated based on a normal distribution of the variables tested. A power of 80% and a bilateral alpha level of 5% were assumed with mean and standard deviation data of a previous study [17]. Thirty-two male Wistar rats (Rattus norvegicus, 35 days old), weighing approximately 100 g, were divided by simple randomization into two groups (*n* = 16/group): the aluminum group and the control group. Then, the rats were allocated to collective plastic cages (4 animals each). During the experimental period, the animals had ad libitum access to water-balanced pelleted food (Presence, Neovia, Brazil), and the temperature-controlled (25 °C) room was maintained under standard 12 h light/dark cycle (lights on at 7:00 am) conditions. The steps of the method are summarized in Figure 5.

### 4.2. Aluminum Exposure Protocol

The aluminum group received 8.3 mg/kg/day of AlCl_3_ diluted in 8 mg/mL distilled water daily for 60 days. The exposure solution was administered by intragastric gavage. The dose of AlCl_3_ used in this study was described in previous studies as a low dose that is capable of promoting changes in biological systems, and corresponds to an animal dose translated from doses of Al to which humans are exposed via diet [10,23]. A previous study using the same experimental model measured the Al levels in animal blood and revealed that the treated animals showed higher Al levels (AlCl_3_: 56.4 ± 3.94 μgL^−1^; control: 24.61 ± 5.21 μgL^−1^, *p* < 0.05) [10]. The control group received only distilled water (H_2_Od) following the same protocol. The animals were weighed weekly to correctly adjust the Al dose.

### 4.3. Sample Collection

After the exposure period, the animals were anaesthetized with an intraperitoneal injection of 10% ketamine hydrochloride (90 mg/kg) and 2% xylazine hydrochloride (9 mg/kg). For eight randomly chosen animals per group, after complete loss of reflexes, the salivary glands were collected and then stored at −80 °C for further biochemistry and proteomic assays. The remaining eight animals per group were perfused for histological analysis. 

### 4.4. Biochemical Assays

Samples were thawed and resuspended in a Tris-HCl solution (20 mM, pH 7.4) for sonic disintegration (~1 g/mL). Then, the total homogenate was used in both the ACAP assay and the LPO assay.

#### 4.4.1. Antioxidant Capacity against Peroxyl Radicals Assay

ACAP was analyzed using the ROS quantitation produced by the equally-concentrated samples (2.5 µg proteins/µL) after exposure to a peroxyl radical generator [59]. Peroxyl radicals were produced by the thermal (35 °C) decomposition of 2,2’-azobis 2 methylpropionamidine dihydrochloride (ABAP; 4 mM; Sigma-Aldrich, St. Louis, MI, USA). For ROS determination, the compound 2’,7’-dichlorofluorescein diacetate (H_2_DCF-DA, Invitrogen™, Whaltan, MA, USA) was used at a final concentration of 40 nM. The readings were carried out in a fluorescence microplate reader (Victor X3, Perkin Elmer, Waltham, MA, USA) every 5 min for 1 h. The relative difference between ROS area with and without ABAP was considered as a measure of antioxidant capacity. From that, the results were transformed to the inverse of the relative area and expressed as % of control.

#### 4.4.2. Lipid Peroxidation Assay

LPO levels in the samples were measured using MDA levels as an indicator [60]. After sonic homogenization, lysates were centrifuged at 3512× *g* for 10 min at 4 °C. Supernatants and standard solutions of MDA were then incubated with a 1:4 solution of methanesulfonic acid:N-methyl-2-phenylindole 10.3 mM diluted in methanol (1:3), at 45 °C for 40 min, followed by spectrophotometric reading (λ = 570 nm). The results are expressed in nanomol per microgram (nmol/µg) of protein and graphed as a percentage of the control. Protein quantification was performed using the Bradford method [61].

### 4.5. Proteomic Analysis

#### 4.5.1. Protein Extraction

Proteomic analysis was performed according to previously described protocols [38,61]. The samples were cryofractured in liquid nitrogen using a cryogenic mill, and soluble proteins were extracted with lysis buffer (7 M urea, 2 M thiourea, diluted in ammonium bicarbonate (AmBic)) under constant stirring at 4 °C. The samples were centrifuged for 30 min at 14,000 rpm at 4 °C, and the total protein concentrations in the supernatants were quantified using Bradford’s method [62]. The assay was carried out in biological triplicate and two samples from different animals were pooled into one. Subsequently, we collected 50 μg of protein from each pooled sample and added AmBic (50 mM) until final concentrations of 1 μg/μL were reached. Quantities of 10 μL of AmBic and 25 μL of 0.2% RapiGEST™ (Waters Co., Manchester, UK) were added and incubated at 37 °C for 30 min, followed by the addition of 2.5 μL of 100 mM dithiothreitol and incubation at 37 °C for 60 min. Then, we added 2.5 μL of iodoacetamide (300 mM); BioRad, Hercules, CA, USA) before incubation for 30 min at room temperature in the dark. Subsequently, 10 μL of trypsin (Thermo Fischer, Waltham, MA, USA) were added and the digestion occurred for 14 h at 37 °C. Furthermore, 10 µL of 5% trifluoroacetic acid (Sigma-Aldrich, St. Louis, MI, USA) was added. The samples were incubated at 90 min at 37 °C, which was followed by posterior centrifugation at 14,000 rpm at 6 °C for 30 min. The supernatants were collected and purified using C18 spin columns (Pierce, Waltham, MA, USA). The samples were resuspended in 12 μL of alcohol dehydrogenase (1 pmol/μL), 108 μL of 3% acetonitrile (Sigma-Aldrich, St. Louis, MI, USA), and 0.1% formic acid (Thermo Fischer, Waltham, MA, USA). 

#### 4.5.2. Mass Spectrometry Analysis

The label-free proteomic analysis was performed in a nanoACQUITY UPLC system (Waters, Milford, MA, USA) coupled to a Xevo Q-TOF G2 mass spectrometer (Waters, Milford, MA, USA). The nanoACQUITY UPLC system was equipped with a Trap Columm (100 Å, 5 μm, 180 μm × 200 mm) and a HSS T3 M-Class type column (analytical column 75 μm × 150 mm; 1.8 μm) (Waters, Milford, MA, USA). The reading and identification of peptides was performed using the ProteinLynx GlobalServer software (PLGS) version 3.03 (Waters, Milford, MA, USA), as previously described [63]. The PLGS software, applying the Monte-Carlo algorithm, was used to determine the difference in protein expression between the groups, considering *p* < 0.05 for downregulated proteins and 1 − *p* > 0.95 for upregulated proteins. The identification of proteins was performed by downloading UniProt databases. Then, bioinformatics analyses were performed using Cytoscape^®^ 3.6 (Java^®^) with the ClusterMarker plugin for the PPI network, and for the determination of the biological process groups based on Gene Ontology annotations, we used the ClueGO plugin [64].

#### 4.5.3. Over-Representation Analysis

A table was generated containing the names of the proteins and their respective Log2Ratio values. For proteins with absolute changes, a value of −1 was adopted when the protein was present only in the control group and 1 was adopted when the protein was present in the exposed group.

For the ORA analysis, R studio software [65] was used with the EGSEA package [66]. In this step, the UNIPROT database was consulted to identify the proteins and the biological processes in which they participate; this database was made available by the Bader Lab. After this verification, we used Cytoscape software [67] with the Enrichment Map plugin to group the sets of proteins that were previously examined, after which the main biological processes were selected for graphic analysis.

Then, a PPI analysis (https://www.networkanalyst.ca/, accessed on 11 November 2021) [68] was performed to generate a representative image containing the top interacting proteins, which were categorized according to the number of protein interactions. The image was generated by R studio software with the GOplot plugin.

### 4.6. Perfusion and Histological Analysis

Eight animals from each group were deeply anaesthetized and then perfused with a saline (NaCl 0.9%) heparinized (1%) solution, followed by a paraformaldehyde 4% buffered solution (0.2 M). The submandibular and parotid glands were removed for histological procedures. The salivary glands were postfixed in 4% formaldehyde until processing. Then, the glands were dehydrated by incubation in solutions with increasing concentrations of ethanol (70%, 80%, 90%, absolute 1, absolute 2), diaphanized in xylol, and embedded in Paraplast (McCormick Scientific, Baltimore, Maryland, EUA). Using a microtome, sections of 5 μm thickness were obtained. These sections were stained with hematoxylin and eosin. 

For the histopathological analysis, the evaluation was performed by two oral pathologists, who were blinded to the treatments, using an optical microscope (Axiophot ZEISS, Hallbergmoos, Germany). This evaluation of the salivary glands was described in a previous study [20], and the following two criteria were used: (1) the relationship between parenchyma (ducts and acini) and stroma (collagen fibers, inflammatory infiltrate, and blood vessels), which describes possible changes in the parenchyma × stroma relationship and (2) possible cellular changes in the glandular parenchyma, defined as atrophy, vacuolization, and/or degranulation.

For the morphometric analysis, images were taken by a colored digital camera coupled to an optic microscope (Leica Microscope DM750, Leica Microsystems^®^, Switzerland, 40× magnification); images of 5 random sagittal sections of the glands, including 5 fields of view of each section, were captured. The total parenchyma area, total stroma area, total acinar area, and total duct area parameters of tissue morphology were evaluated and expressed in μm^2^ [69]. The values were obtained using the ImageJ software (NIMH, NIH, Bethesda, MD, USA) digital image analyzer.

### 4.7. Statistical Analysis

The data obtained in this study were plotted using GraphPad Prism 7.0 software (San Diego, CA, USA). The values obtained from the oxidative biochemical assays and histological analysis were evaluated for normality with the Shapiro–Wilk method and then compared using the Student’s *t*-test. The significance level was *p* < 0.05. The results are expressed as the mean ± standard error of the mean (SEM). The test power was calculated based the differences between the group averages with OpenEpi software (Version 2.3.1), considering a type-I error of 5% and a power of 80% (for all values, see Table S3). In the proteomic analysis, the PLGS software was used to obtain the differences in protein expression between the groups, applying the Monte-Carlo algorithm (*p* < 0.05 for downregulated proteins and 1 − *p* > 0.95 for upregulated proteins).

## 5. Conclusions

For the first time, changes in the proteome profile of parotid and submandibular glands after low- and long-term Al exposure were demonstrated. These changes were associated with glandular oxidative stress and morphological impairments. We showed different responses by the glands due to their different characteristics, but all the results led to the suggestion that Al exert harmful effects on these organs. More studies are needed to better understand the toxic effect of Al on salivary glands and the potential functional failure outcomes.

## Figures and Tables

**Table 1:** Morphometric measurements of the rats’ salivary glands after long-term exposure to aluminum, *n* = 8/group (mean ± SEM).

Measures	Parotid Glands			Submandibular Glands		
	Control Group	Aluminum Group	*p* Value *	Control Group	Aluminum Group	*p* Value *
Total parenchyma area (µm^2^)	64,563 ± 2613	58,262 ± 1100	0.0397	69,726 ± 1713	54,543 ± 1564	0.0001
Total stroma area (µm^2^)	21,980 ± 634	28,803 ± 734.7	0.0011	4941 ± 1099	24,263 ± 1473	<0.0001
Total acinar area (µm^2^)	62,676 ± 3062	55,020 ± 426.7	0.0326	66,660 ± 2079	50,606 ± 1911	0.0006
Total ductal area (µm^2^)	2325 ± 611.8	2159 ± 279.7	0.7852	3002 ± 285.9	4022 ± 255.2	0.0312

* (Student’s *t* test, *p* < 0.05).

## Data Availability

All data are available within the article and in Supplementary Materials.

## Supplementary Materials

The following supporting information can be downloaded at: https://www.mdpi.com/article/10.3390/ijms23042251/s1.
