# Supplementary material for: Salivary Glands after Prolonged Aluminum Exposure: Proteomic Approach Underlying Biochemical and Morphological Impairments in Rats
Source: Int J Mol Sci. 2022 Feb 18;23(4):2251. doi: 10.3390/ijms23042251 (PMC8877476; doi:10.3390/ijms23042251)
Supplement: Supplementary file 1 [file ijms-23-02251-s001.zip › ijms-1555459-supplementary.pdf]

International Journal of
Molecular Sciences

MDPI

Supplementary Materials

Salivary glands after prolonged aluminum exposure: proteomic approach underlying biochemical and morphological impairments in rats

Deiweson Souza-Monteiro, Márcia Cristina dos Santos Guerra, Leonardo Oliveira Bittencourt, Walessa Alana Bragança Aragão, Aline Dionizio, Felipe Martins Silveira, Marília Afonso Rebelo Buzalaf, Manoela Domingues Martins, Maria Elena Crespo-Lopez, Rafael Rodrigues Lima

Table S1. Identified proteins with significant different status of regulation in the parotid glands of rats exposed to aluminum (Exposed group vs. Control group)

**Accession ID[a]**	**Protein Name**	***PLGS* Score**	**Fold Change**
**P47942**	Dihydropyrimidinase-related protein 2	67.84	15.64
**P54708**	Potassium-transporting ATPase alpha chain 2	32.04	14.15
**Q64541**	Sodium/potassium-transporting ATPase subunit alpha-4	32.04	14.15
**Q05511**	Serine protease hepsin	126.6	5.47
**P07335**	Creatine kinase B-type	161.62	5.16
**Q68FR8**	Tubulin alpha-3 chain	1011.09	5.16
**P50398**	Rab GDP dissociation inhibitor alpha	56.32	5.05
**Q6P9V9**	Tubulin alpha-1B chain	1190.77	4.85
**Q9Z2L0**	Voltage-dependent anion-selective channel protein 1	68.45	4.57
**Q29RW1**	Myosin-4	20.35	4.01
**P00564**	Creatine kinase M-type	69.39	3.9
**P48675**	Desmin	38.96	3.9
**P11980**	Pyruvate kinase PKM	183.96	3.42
**P35704**	Peroxiredoxin-2	130.15	3.35
**P50399**	Rab GDP dissociation inhibitor beta	65.89	3.32
**P09117**	Fructose-bisphosphate aldolase C	199.56	3.22
**O35412**	Signal-induced proliferation-associated 1-like protein 1	294.34	3.19
**P07632**	Superoxide dismutase [Cu-Zn]	148.7	3.16
**P00507**	Aspartate aminotransferase, mitochondrial	95.88	3.13

**P10888**	Cytochrome c oxidase subunit 4 isoform 1, mitochondrial	117.42	3.1
**P11442**	Clathrin heavy chain 1	46.33	2.8
**P05065**	Fructose-bisphosphate aldolase A	71.84	2.77
**O88989**	Malate dehydrogenase, cytoplasmic	171.18	2.69
**P00406**	Cytochrome c oxidase subunit 2	529.24	2.59
**P0DP30**	Calmodulin-2	166.25	2.56
**P16617**	Phosphoglycerate kinase 1	76.33	2.56
**P0DP29**	Calmodulin-1	166.25	2.53
**P0DP31**	Calmodulin-3	166.25	2.51
**P11240**	Cytochrome c oxidase subunit 5A, mitochondrial	277.53	2.51
**P35213**	14-3-3 protein beta/alpha	125.06	2.48
**P68511**	14-3-3 protein eta	125.06	2.48
**P68255**	14-3-3 protein theta	125.06	2.48
**P61983**	14-3-3 protein gamma	125.06	2.46
**O70417**	Prolactin-inducible protein homolog	2677.87	2.46
**P48500**	Triosephosphate isomerase	355.39	2.44
**P21704**	Deoxyribonuclease-1	3766.11	2.41
**P62260**	14-3-3 protein epsilon	206.65	2.29
**P63102**	14-3-3 protein zeta/delta	419.94	2.23
**P45592**	Cofilin-1	380.9	2.16
**P11030**	Acyl-CoA-binding protein	720.64	2.16
**P04636**	Malate dehydrogenase, mitochondrial	364.74	2.05
**P06686**	Sodium/potassium-transporting ATPase subunit alpha-2	57.33	2.03
**P06687**	Sodium/potassium-transporting ATPase subunit alpha-3	57.33	2.01
**Q9ER34**	Aconitate hydratase, mitochondrial	66.53	1.95
**P17764**	Acetyl-CoA acetyltransferase, mitochondrial	117.63	1.86
**Q9ESV6**	Glyceraldehyde-3-phosphate dehydrogenase, testis-specific	86.11	1.82
**P06685**	Sodium/potassium-transporting ATPase subunit alpha-1	68.7	1.72

**O88767**	Protein/nucleic acid deglycase DJ-1	251.68	1.68
**P15429**	Beta-enolase	33.13	1.65
**Q05962**	ADP/ATP translocase 1	57.97	1.63
**P63029**	Translationally-controlled tumor protein	700.39	1.63
**P50137**	Transketolase	74.27	1.6
**P19944**	60S acidic ribosomal protein P1	599.01	1.57
**Q06647**	ATP synthase subunit O, mitochondrial	76.99	1.57
**P04797**	Glyceraldehyde-3-phosphate dehydrogenase	1694.24	1.54
**Q5XI73**	Rho GDP-dissociation inhibitor 1	105.87	1.54
**P04642**	L-lactate dehydrogenase A chain	110.59	1.52
**Q6AY56**	Tubulin alpha-8 chain	775.22	1.52
**Q63471**	BPI fold-containing family A member 2	3098.57	1.51
**P56574**	Isocitrate dehydrogenase [NADP], mitochondrial	123.58	1.48
**Q6RY07**	Acidic mammalian chitinase	10981.75	1.46
**Q9WTT6**	Guanine deaminase	108.74	1.43
**P15999**	ATP synthase subunit alpha, mitochondrial	460.83	1.4
**P38983**	40S ribosomal protein AS	409.09	1.38
**P04764**	Alpha-enolase	511.54	1.36
**P63018**	Heat shock cognate 71 kDa protein	775.94	1.35
**P62083**	40S ribosomal protein S7	477.82	1.32
**P06761**	Endoplasmic reticulum chaperone BiP	1280.33	1.32
**Q6P9T8**	Tubulin beta-4B chain	878.95	1.31
**Q5XIF6**	Endoplasmin	397.33	1.3
**P62630**	Elongation factor 1-alpha 1	2887.93	1.28
**Q66HD0**	Tubulin alpha-4A chain	993.58	1.26
**P04906**	Glutathione S-transferase P	242.17	1.25
**Q63716**	Peroxiredoxin-1	264.94	1.25
**P85108**	Tubulin beta-2A chain	818.22	1.25

**Q4QRB4**	Tubulin beta-3 chain	451.9	1.25
**P18418**	Calreticulin	120.36	1.22
**P68370**	Tubulin alpha-1A chain	1194.16	1.22
**Q3KRE8**	Tubulin beta-2B chain	814.85	1.22
**P69897**	Tubulin beta-5 chain	915.07	1.22
**P63039**	60 kDa heat shock protein, mitochondrial	74.89	1.21
**P12020**	Cysteine-rich secretory protein 1	7698.82	1.21
**P0DMW0**	Heat shock 70 kDa protein 1A	336.58	1.21
**Q64057**	Alpha-aminoadipic semialdehyde dehydrogenase	207.59	1.2
**P62632**	Elongation factor 1-alpha 2	1272.4	1.2
**P0DMW1**	Heat shock 70 kDa protein 1B	336.58	1.2
**P55063**	Heat shock 70 kDa protein 1-like	336.58	1.2
**P04785**	Protein disulfide-isomerase	341.37	1.19
**P10719**	ATP synthase subunit beta, mitochondrial	915.78	1.17
**Q6AYZ1**	Tubulin alpha-1C chain	1062.01	1.15
**P10111**	Peptidyl-prolyl cis-trans isomerase A	2191.43	1.11
**Q6P6Q2**	Keratin, type II cytoskeletal 5	133.48	-0.51
**P00689**	Pancreatic alpha-amylase	1733.1	-0.52
**P32551**	Cytochrome b-c1 complex subunit 2, mitochondrial	39.23	-0.54
**Q6LED0**	Histone H3.1	774.82	-0.6
**P84245**	Histone H3.3	774.82	-0.6
**P02466**	Collagen alpha-2(I) chain	138.73	-0.62
**P0C0S7**	Histone H2A.Z	3302.7	-0.66
**Q6IE52**	Murinoglobulin-2	97.41	-0.66
**P02091**	Hemoglobin subunit beta-1	16061.07	-0.7
**P11517**	Hemoglobin subunit beta-2	3174.66	-0.7
**Q63279**	Keratin, type I cytoskeletal 19	191.73	-0.71
**Q64119**	Myosin light polypeptide 6	303.88	-0.71

**P02262**	Histone H2A type 1	10690.25	-0.72
**P0C169**	Histone H2A type 1-C	10690.25	-0.72
**P0C170**	Histone H2A type 1-E	10690.25	-0.72
**Q4FZT6**	Histone H2A type 3	10690.25	-0.72
**A9UMV8**	Histone H2A.J	10690.25	-0.72
**P68035**	Actin, alpha cardiac muscle 1	7131.74	-0.73
**P68136**	Actin, alpha skeletal muscle	7057.67	-0.73
**P62738**	Actin, aortic smooth muscle	6437.13	-0.73
**P63269**	Actin, gamma-enteric smooth muscle	6437.13	-0.73
**Q64598**	Histone H2A type 1-F	10690.25	-0.73
**P0CC09**	Histone H2A type 2-A	10690.25	-0.73
**Q00728**	Histone H2A type 4	10690.25	-0.73
**P60711**	Actin, cytoplasmic 1	14760.75	-0.74
**P63259**	Actin, cytoplasmic 2	14760.75	-0.74
**P02770**	Serum albumin	10533.53	-0.74
**Q00729**	Histone H2B type 1-A	2677.81	-0.75
**Q5BJY9**	Keratin, type I cytoskeletal 18	1256.79	-0.76
**P0CG51**	Polyubiquitin-B	517.5	-0.78
**Q10758**	Keratin, type II cytoskeletal 8	2152.34	-0.79
**Q63429**	Polyubiquitin-C	517.5	-0.79
**P12346**	Serotransferrin	580.1	-0.79
**P62982**	Ubiquitin-40S ribosomal protein S27a	517.5	-0.79
**P62986**	Ubiquitin-60S ribosomal protein L40	517.5	-0.79
**P20760**	Ig gamma-2A chain C region	1255.75	-0.82
**P01946**	Hemoglobin subunit alpha-1/2	25408.2	-0.84
**Q00715**	Histone H2B type 1	14623.03	-0.89
**P62804**	Histone H4	12183.1	-0.89
**Q641X9**	39S ribosomal protein L9, mitochondrial	122.25	-

**Q9JKL8**	Activity-dependent neuroprotector homeobox protein	40.46	-
**O89046**	Coronin-1B	66.86	-
**Q9JM01**	Cyclin-dependent kinase-like 3	47.42	-
**O35964**	Endophilin-A2	82.8	-
**Q499U2**	Engulfment and cell motility protein 3	47.63	-
**P31977**	Ezrin	31.89	-
**Q00960**	Glutamate receptor ionotropic, NMDA 2B	28.32	-
**D3ZIZ1**	GRAM domain-containing 2A	66.87	-
**D4A3K5**	Histone H1.1	38.92	-
**P15865**	Histone H1.4	177.24	-
**P06349**	Histone H1t	177.24	-
**P20762**	Ig gamma-2C chain C region	63.41	-
**P01836**	Ig kappa chain C region, A allele	653.96	-
**P20767**	Ig lambda-2 chain C region	93.45	-
**F1LZ52**	Kelch-like protein 3	55.87	-
**Q6IMF3**	Keratin, type II cytoskeletal 1	79.27	-
**Q6IG01**	Keratin, type II cytoskeletal 1b	79.27	-
**Q4FZU2**	Keratin, type II cytoskeletal 6A	79.27	-
**Q6IG04**	Keratin, type II cytoskeletal 72	79.27	-
**Q6AYL9**	Kinetochore protein Nuf2	48.53	-
**P30349**	Leukotriene A-4 hydrolase	70.99	-
**P30919**	N(4)-(Beta-N-acetylglucosaminyl)-L-asparaginase	65.27	-
**O35260**	Nucleus accumbens-associated protein 1	30.54	-
**P24368**	Peptidyl-prolyl cis-trans isomerase B	108.87	-
**P21807**	Peripherin	61.32	-
**Q63945**	Protein SET	206.16	-
**P81128**	Rho GTPase-activating protein 35	56.27	-
**Q62868**	Rho-associated protein kinase 2	46.84	-

**Q9Z220**	Testis-specific gene 10 protein	29.82	-
**B5DEL3**	Tetratricopeptide repeat protein 17	39.23	-
**P63312**	Thymosin beta-10	498.09	-
**Q9EQS0**	Transaldolase	128.06	-
**Q5U2W5**	Transducin beta-like protein 3	49.32	-
**Q64350**	Translation initiation factor eIF-2B subunit epsilon	58.14	-
**D3ZFH6**	UBX domain-containing protein 440S ribosomal protein S3a	56.11	-

[a]Accession ID according to Uniprot.org database. Positive and negative values of fold change, indicate up and downregulated proteins, respectively. Results of the comparison between aluminum group and control group. Signs of - indicate absence expression in the exposed group.

Table S2. Identified proteins with significant different status of regulation in the submandibular glands of rats exposed to aluminum (Exposed group vs. Control group)

**Accession ID[a]**	**Protein Name**	***PLGS* Score**	**Fold Change**
**P36375**	Glandular kallikrein-10	374.62	1.43
**P07647**	Submandibular glandular kallikrein-9	321.87	1.42
**P60711**	Actin, cytoplasmic 1	153.3	1.14
**P63259**	Actin, cytoplasmic 2	153.3	1.14
**P02091**	Hemoglobin subunit beta-1	571.39	-0.55
**P11517**	Hemoglobin subunit beta-2	447.55	-0.59
**P01946**	Hemoglobin subunit alpha-1/2	1091.38	-0.6
**Q64598**	Histone H2A type 1-F	421.36	-0.69
**P0C170**	Histone H2A type 1-E	421.36	-0.7
**Q4FZT6**	Histone H2A type 3	421.36	-0.7
**Q00728**	Histone H2A type 4	421.36	-0.7
**P62804**	Histone H4	384.08	-0.75
**O70417**	Prolactin-inducible protein homolog	781.02	-0.88
**P62246**	40S ribosomal protein S15a	75.79	+

**P04644**	40S ribosomal protein S17	533.72	+
**P62083**	40S ribosomal protein S7	130.26	+
**P38983**	40S ribosomal protein SA	232.16	+
**P63039**	60 kDa heat shock protein, mitochondrial	51.94	+
**P19944**	60S acidic ribosomal protein P1	741.43	+
**P23358**	60S ribosomal protein L12	105.16	+
**P17764**	Acetyl-CoA acetyltransferase, mitochondrial	130.35	+
**Q9ER34**	Aconitate hydratase, mitochondrial	46.82	+
**P11030**	Acyl-CoA-binding protein	279.62	+
**P15429**	Beta-enolase	89.14	+
**P18418**	Calreticulin	69.26	+
**P18886**	Carnitine O-palmitoyltransferase 2, mitochondrial	39.64	+
**P02454**	Collagen alpha-1(I) chain	38.15	+
**P25809**	Creatine kinase U-type, mitochondrial	235.38	+
**P07153**	Dolichyl-diphosphooligosaccharide--protein glycosyltransferase subunit 1	32.19	+
**Q5XIL8**	Dynein intermediate chain 1, axonemal	50.59	+
**P13803**	Electron transfer flavoprotein subunit alpha, mitochondrial	58.82	+
**Q68FU3**	Electron transfer flavoprotein subunit beta	115.73	+
**P05197**	Elongation factor 2	92.56	+
**Q66HD0**	Endoplasmin	49.99	+
**P07323**	Gamma-enolase	52.23	+
**P09606**	Glutamine synthetase	30.17	+
**P04797**	Glyceraldehyde-3-phosphate dehydrogenase	377.03	+
**P63018**	Heat shock cognate 71 kDa protein	62.79	+
**P14659**	Heat shock-related 70 kDa protein 2	37.44	+
**Q6LED0**	Histone H3.1	183.65	+
**P84245**	Histone H3.3	183.65	+
**P5674**	Isocitrate dehydrogenase [NADP], mitochondrial	109.56	+

**Q6IG12**	Keratin, type II cytoskeletal 7	202.32	+
**O88989**	Malate dehydrogenase, cytoplasmic	119.16	+
**Q9R063**	Peroxiredoxin-5, mitochondrial	118.02	+
**P62963**	Profilin-1	278.67	+
**P11598**	Protein disulfide-isomerase A3	58.71	+
**Q62902**	Protein ERGIC-53	139.98	+
**P11980**	Pyruvate kinase PKM	45.9	+
**Q4QR99**	Queuine tRNA-ribosyltransferase catalytic subunit 1	59.01	+
**P50398**	Rab GDP dissociation inhibitor alpha	32.99	+
**P50399**	Rab GDP dissociation inhibitor beta	42.49	+
**Q6NYB7**	Ras-related protein Rab-1A	174.7	+
**Q5XI73**	Rho GDP-dissociation inhibitor 1	210.91	+
**P13432**	SMR1 protein	299.2	+
**Q66X93**	Staphylococcal nuclease domain-containing protein 1	60.78	+
**P68370**	Tubulin alpha-1A chain	122.88	+
**Q6P9V9**	Tubulin alpha-1B chain	122.88	+
**Q6AYZ1**	Tubulin alpha-1C chain	122.88	+
**Q68FR8**	Tubulin alpha-3 chain	83.38	+
**Q5XIF6**	Tubulin alpha-4A chain	54.94	+
**F1LNJ2**	U5 small nuclear ribonucleoprotein 200 kDa helicase	20.79	+
**Q6IG05**	Keratin, type II cytoskeletal 75	36.89	-

[a]Accession ID according to Uniprot.org database. Positive and negative values of fold change, indicate up and downregulated proteins, respectively. Results of the comparison between aluminum group and control group. Signs of + and - indicate exclusive or absence expression in the exposed group, respectively.

International Journal of *Molecular Sciences*

MDPI

Table S3. Description of all analysis values of the study.

Analyze	Description of unit	Mean (Control)	Mean ($AlCl_3$)	SD (Control)	SD ($AlCl_3$)	SEM (Control)	SEM ($AlCl_3$)	Power (1-β err prob)
**ACAP - Parotid gland**	% of control	100	57.79	23.79	15.16	8.993	5.73	98.85%
**LPO - Parotid gland**	% of control	100	140.4	15.87	22.07	5.612	9.011	98.76%
**ACAP - Submandibular gland**	% of control	100	36.9	21.64	21.01	8.179	7.941	100%
**LPO - Submandibular gland**	% of control	100	194.1	31.79	41.4	15.89	23.9	99.91%
**Parenchyma - Parotid gland**	µm²	64563	58262	4525	2460	2613	1100	93.32%
**Stroma - Parotid gland**	µm²	21980	28803	1098	1469	634.2	734.7	100%
**Acini - Parotid gland**	µm²	62676	55020	5304	853.3	3062	426.7	98.08%
**Ducts - Parotid gland**	µm²	2325	2159	1060	625.5	611.8	279.7	5.367%
**Parenchyma - Submandibular gland**	µm²	69726	54543	3830	3830	1713	1564	100%
**Stroma - Submandibular gland**	µm²	4941	24263	2457	3607	1099	1473	100%
**Acini - Submandibular gland**	µm²	66660	50606	4158	4682	2079	1911	100%
**Ducts - Submandibular gland**	µm²	3002	4022	571.9	625.1	285.9	255.2	92.68%

Table S3: Oxidative biochemistry assays (ACAP: Antioxidant Capacity Against Peroxyl Radicals and LPO: Lipid Peroxidation) and Morphometric analysis (Parenchyma, stroma, acini and ducts areas) values of the experimental study. Results are expressed as mean, SD: Standard deviation, SEM: Standard error of mean and Test Power (1-β error probability).
